# Religious aspects of assisted reproduction

**Published:** 2016-03-28

**Authors:** HN Sallam, NH Sallam

**Affiliations:** Department of Obstetrics and Gynaecology and Alexandria Fertility; and IVF Center, Alexandria, Egypt.

**Keywords:** Assisted reproduction, Buddhism, Christianity, Hinduism, Islam, ICSI, IVF, Judaism, religion, religious aspects

## Abstract

Human response to new developments regarding birth, death, marriage and divorce is largely shaped by religious beliefs. When assisted reproduction was introduced into medical practice in the last quarter of the twentieth century, it was fiercely attacked by some religious groups and highly welcomed by others. Today, assisted reproduction is accepted in nearly all its forms by Judaism, Hinduism and Buddhism, although most Orthodox Jews refuse third party involvement. On the contrary assisted reproduction is totally unacceptable to Roman Catholicism, while Protestants, Anglicans, Coptic Christians and Sunni Muslims accept most of its forms, which do not involve gamete or embryo donation. Orthodox Christians are less strict than Catholic Christians but still refuse third party involvement. Interestingly, in contrast to Sunni Islam, Shi’a Islam accepts gamete donation and has made provisions to institutionalize it. Chinese culture is strongly influenced by Confucianism, which accepts all forms of assisted reproduction that do not involve third parties. Other communities follow the law of the land, which is usually dictated by the religious group(s) that make(s) the majority of that specific community. The debate will certainly continue as long as new developments arise in the ever-evolving field of assisted reproduction.

## Introduction

Religion is a cultural system of behaviours and practices, world views, ethics, and social organiza­tion that relate humanity to an order of existence. About 84% of the world’s population is affiliated with one of the five largest religions namely Christianity, Islam, Hinduism, Buddhism or folk religion (Pew Research Center, 2015; Stonawski et al., 2015; Hackett et al., 2015; Wikipedia, 2016).

The birth of Louise Brown, the first baby born from the technique of in­-vitro fertilization, on the 25th of July 1978 presented the world with the sobering fact of the possibility of achieving pregnancy and birth through methods previously unthinkable. Like many overwhelming achievements, many people passed through the stages of denial, confusion and finally acceptance. When all the dust settled, these people started to react to this new reality and their reaction was mainly determined by their religious beliefs, where people usually look for answers to questions of birth, death, marriage or divorce. This article discusses how religion shaped the reaction of various communities around the world to assisted reproduction.

## World religions

According to a Pew report published in 2012, more than eight­-in-­ten people in the world identify with a religious group. A comprehensive demographic study of more than 230 countries and territories conducted by the Pew Research Center’s Forum on Religion and Public Life estimated that there are 5.8 billion religiously affiliated adults and children around the globe, representing 84% of the 2010 world population of 6.9 billion. The remaining 1.1 billion (16%) were not affiliated to any religion (Pew Research Center, 2012).

Of those religiously affiliated, 2.2 billion are Christians (32% of the world’s population), 1.6 billion Muslims (23%), 1 billion Hindus (15%), 500 million Buddhists (7%) and 14 million Jews (0.2%). In addition, more than 400 million people (6%) practice various folk or traditional religions, including African traditional religions, Chinese folk religions, Native American religions and Australian aboriginal religions. An estimated 58 million people – slightly less than 1% of the global population – belong to other religions, including the Baha’i faith, Jainism, Sikhism, Shintoism, Taoism, Tenrikyo, Wicca and Zoroastrianism (Pew Research Center, 2012). Table I shows the distribution of the major world religions (Pew Research Center, 2012).

**Table I:** — Pre-operative baseline characteristics in patients that were operated because of endometrial cancer in this study (laparoscopy = group I; conversion = group II; laparotomy invalid = group IIIa; laparotomy valid = group IIIB).

Religion	2010 population	% of world population
Christians	2,168,330,000	31.4%
Muslims	1,599,700,000	23.2%
Unaffiliated	1,131,150,000	31.4%
Christians	2,168,330,000	16.4%
Hindus	1,032,210,000	15.0%
Buddhists	487,760,000	7.1%
Folk Religions	404,690,000	5.9%
Other Religions	58,150,000	0.8%
Jews	13,860,000	0.2%
World total	6,895,850,000	100.0%

## Assisted reproduction in Judaism

The 14 million people around the world that identify themselves as Jewish represent 0.2% of the global population. Geographically, Jews are concentrated primarily in North America (44%) and the Middle East­-North Africa region (41%). The remainder of the global Jewish population is found in Europe (10%), Latin America and the Caribbean (3%), Asia and the Pacific (between 1% and 2%) and sub-Saharan Africa (less than 1%).

**Fig. 1:**
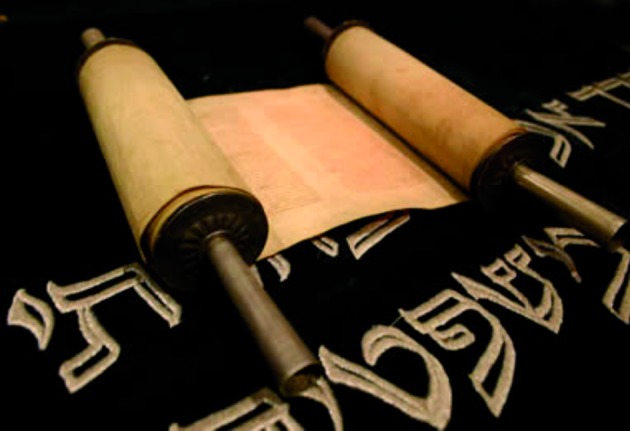
— The Torah or the Pentateuch is the central reference of the religious Judaic tradition and includes the first five books of the twenty-four books of the Tanakh. The term Torah means instruction and offers a way of life for those who follow it.

Followers of the Jewish faith are encouraged to have children. In the Torah, people are instructed to “Be fruitful and multiply, fill the earth and subdue it” (Genesis 1: 28) (Fig. 1). Infertility is also a recurring theme in the Jewish traditions. Three out of four Biblical matriarchs suffered from infertility: Sarah resigned herself to not having children and even laughed at the possibility of pregnancy at her age, but was eventually pregnant and bore Isaac. Rebecca was more positive and asked Isaac to interfere on her behalf and his prayers were answered. On the other hand, Rachel, wife of Jacob told her husband in desperation: “Give me children, otherwise I am dead”. She then resorted to extreme measures. Her nephew (eldest son of Leah) had brought to his mother “dudaim” a plant known to enhance fertility (Genesis 30: 14). Rachel begged her sister for the plant and made a deal with her: she would allow her to spend one night with Jacob in return for the plant, a relation that led to the birth of Lea’s fifth-born son. Rachel was finally “remembered” by God. She conceived and bore Joseph and kept saying afterwards: “God has taken away my disgrace” (Genesis 30: 23).

Today, three sects of Judaism exist: (1) the Orthodox Jews who form about 10%, (2) the conservative Jews, who form about 5% and (3) the reformed Jews who form the majority at about 85% (Silber, 2008). In matters of birth, death, marriage and divorce, all Jews follow the Ten Commandments given by God to Moses on Mount Sinai, but the three sects differ in their acceptance of various aspects of assisted reproduction. So, in general, if IVF is mandatory, it is allowed as Jews have an obligation to “be fruitful and multiply”. PGD and PGS are also allowed because the soul does not enter the body until 40 days (Halacha = Kosher). Selective reduction is acceptable as it enhances the possibility of life as determined by doctors. Embryo research to promote life is also acceptable as well as therapeutic cloning and any research, which can promote life­saving treatment (e.g. stem cell and cellular replacement therapy) (Silber, 2008).

However, some aspects of assisted reproduction are still controversial and the three sects of Judaism differ in their acceptance. For example, semen collection for any form of assisted reproduction is still a matter of debate within the Jewish faith. Orthodox Rabbis insist that spilling of the seed is forbidden and consequently, the husband may not ejaculate to provide a specimen. Special devices have been invented to prevent spillage of the seeds (non­medicated condoms) and are accepted by some Rabbis as a permissible method for obtaining the sperm. More liberal Rabbis permit ejaculation on the basis that the intention is to enhance procreation. Obtaining spermatozoa from men with azoospermia has also been a debatable point. In fact, the Talmud specifically forbids “cutting the sperm ducts” but as Jews have to “be fruitful and multiply”, obtaining spermatozoa through the techniques of TeSE and MESA are allowed and even mandatory because the first commandment takes priority over the Talmud. This is called “Leniency”, a condition which arises when the understandable Rabbis accommodate the medical necessities within the religious text.

Artificial insemination using the husband’s sperm is also allowed by all Jewish sects as long as the sperm is not wasted and again, special devices are recommended for sperm collection (non­medicated condoms), but donor insemination is not allowed by most Rabbis. However, some Rabbis are more lenient than others (e.g. Rabbi Moshe Tendler) and the late Rabbi Moshe Feinstein, probably the most respected Orthodox Rabbis of the 20th century, felt that sperm donation is a private matter for the couple to decide (Silber, 2008). If donor insemination is allowed and since Jewishness is conferred through the mother’s line, most conservative Rabbis prefer a non­-Jewish sperm donor to prevent adultery between a Jewish man and a Jewish woman and to prevent future genetic incest among the offspring of anonymous donors (Kahn, 2000; Schenker, 2005; Wahrzman, 2009). Similarly, most Rabbis do not allow oocyte or embryo donation. They do not consider it adultery but strongly discourage the practice. In addition, if the genetic mother is not Jewish, the child cannot be Jewish, at least according to Rabbi Moshe Heinemann. However, recently some Rabbis decided to permit egg donation with oocytes of non­-Jewish donors to prevent possible incest among the offspring, explaining that Jewishness will be conferred to the child by the religion of the parturient (Ron-­El and Rizk, 2012).

Embryo freezing for replacement in the future is also permissible (halacha). Spare embryos left afterwards can be passively destroyed (e.g. by thawing) but active destruction is not allowed. Similarly, using the embryos for research and then destroying them is not permissible (Silber, 2008). Sex (gender) selection is also allowed for couples who have at least four children of the same sex, and also for some religious indications (e.g. when an azoospermic man from the clan of Kohen, a Kohanim, is using donor sperm to impregnate his wife). He must not have a son, as this son will not be able to perform the religious duties of the Kohanim) (Ron­-El and Rizk, 2012).

Another debatable point within the Jewish faith is surrogacy, as most Rabbis do not accept it. However, for the Rabbis who allow it, single Jewish women are preferred as surrogates, both to avoid the implications of adultery for married surrogate women and to confer Jewishness through a Jewish woman’s gestation of the foetus (Silber, 2008). These controversies within the Jewish state concerning IVF mean that the couples may sometime have to search for the “right Rabbi” for advice. Finally, in IVF clinics dealing with orthodox Jewish patients, orthodox women called *“maschigots”*, are present in the clinics. They observe the laboratory technicians to make sure that the correct sperm and correct eggs are being united – so as not to produce a *mamzer*, or an illegitimate child (Kahn, 2000).

## Assisted reproduction in Christianity

**Fig. 2:**
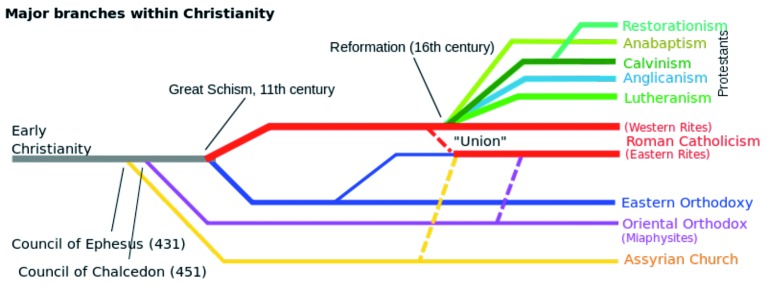
— Major branches of Christianity (By Hogweard ­ File: Christianity Branches.svg, Public Domain, https://commons.wikimedia.org/w/index.php?curid=20414004).

Christians form about 31.5 % of the world population (2.2 billions). A number of various churches exist within Christianity and each one of them reacted differently to assisted reproduction. Most of these churches are opposed to assisted reproduction, although some of them are more fiercely opposed than others. The major branches of Christianity are shown in Figure 2.

According to the Pew Report published in 2011, about half of all Christians worldwide are Catholic (50%), while more than a third are Protestant (37%). Orthodox communions comprise 12% of the world’s Christians. Other Christian groups, which make up the remaining 1%, include the Church of Jesus Christ of Latterday Saints (Mormons), Jehovah’s Witnesses and the Christian Science Church (Pew Research Center, 2011) (Table II).

**Table II:** — Distribution of various Christian denominations (Pew Research Center’s Forum on Religion & Public Life, Global Christianity, December 2011).

Denomination	Number	Percentage of world population	Percentage of Christian population
Catholic	1,094,610,000	15.9%	50.1%
Protestants	800,640,000	11.6%	36.7%
Orthodox	260,380,000	3.85	11.9%
Other Christian	28,430,000	0.4%	1.3%
Total Christian	2,184,060,000	31.7%	100.0%

## Assisted reproduction in the Catholic Church

**Fig. 3:**
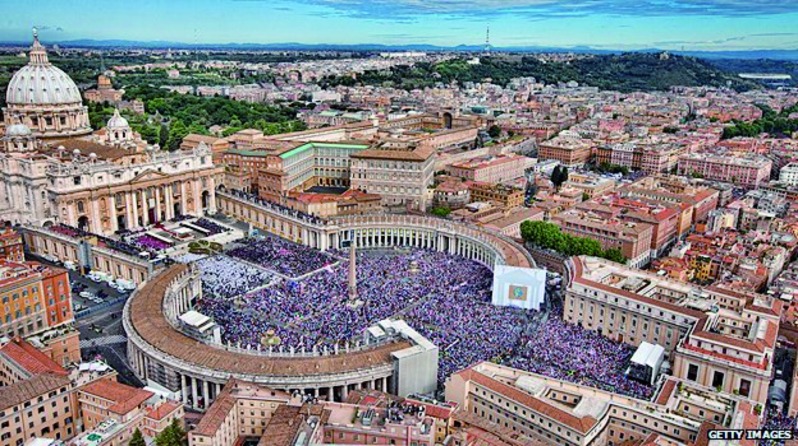
— St Peter’s Basilica is the seat of the Roman Catholic Church, the largest Christian church, with more than 1.2 billion members worldwide. It is headed by the Bishop of Rome known as the Pope. Its doctrines are summarized in the Nicene Creed and it is also notable within the Western Christian tradition for its celebration of the seven sacraments.

The Roman Catholic Church continued as the mainline Church from early Christianity until today. It is headed by the Roman Catholic Pope who sits on the throne of St. Peter in Rome (Fig. 3). There are an estimated 1.2 billion Roman Catholics in the world, according to Vatican figures. More than 40% of the world’s Catholics live in Latin America -­ but Africa has seen the biggest growth in Catholic congregations in recent years (Pew Research Center, 2011).

The main laws of the Catholic Church come from (1) the Holy Book i.e. the Bible and (2) the Church’s traditions, which come from Church’s decisional boards, priests and dogmatic teachings (Abdallah, 2008). Accordingly, the 3 leading principles related to the family, the child and reproduction are (1) the protection of the human being from the moment of its conception, (2) that the child is the fruit of marriage, as God commends husband and wife to have children, and (3) that integrity and dignity norms must be taken into consideration in all these matters (Abdallah, 2008).

The position of the Catholic Church regarding assisted reproduction follows the proclamation issued in 1956 by Pope Pius XII who defined artificial fecundation as immoral and illegal, because it separates procreation and sexual normal function (Pope Pius XII, 1956). These views were reinforced by Pope Paul VI in 1968 and again in the report issued by the Roman Catholic Church in 1987 entitled “Respect for Human Life and the Dignity of Procreation” which stated that “Children are a gift and a blessing from God and that although science makes some things possible it does not make them right. Research must continue into the causes of infertility, but the morality of these should be carefully considered” (Pope Paul VI, 1968; Roman Catholic Church, 1989). Consequently, all forms of assisted reproduction including IUI, IVF, ICSI, ET and surrogate motherhood are not accepted. IUI can be accepted if the semen is collected by sexual intercourse while AID is forbidden because it involves a third party. Moreover, the Catholic Church offers its respect and protection to the human being starting with its first seconds of existence; it therefore considers the zygote, pre­-embryo, embryo and foetus as persons and strongly disapproves research on embryos, cryopreservation and abortion (Abdallah, 2008).

## Protestantism and assisted reproduction

**Fig. 4:**
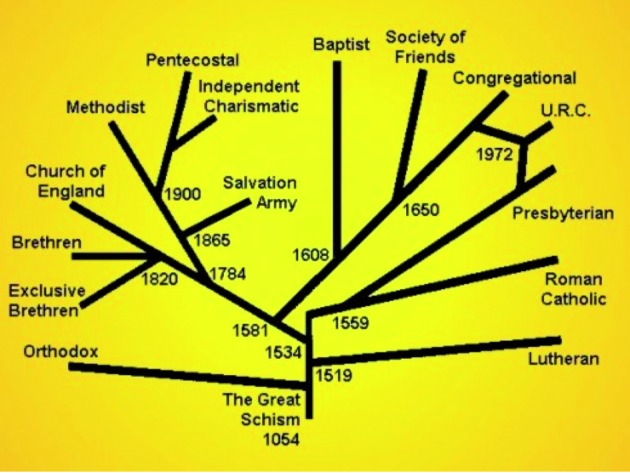
— Various Protestant Churches with their date of establishment (http://worldreligions.weebly.com/christianity.html).

The beginning of the sixteenth century showed significant discontent with the Roman Catholic Church in some parts of Europe and in 1517 Martin Luther nailed his famous 95 Theses to the door of the church in Wittenburg challenging the authority of the Catholic Church. This was followed by similar acts of dissent in other parts of Europe creating various Christian sects collectively known as Protestants, each with its own Church. Today the main protestant denominations are the Lutherans, the Calvinists (including the Presbyterians and the Congregationalists), the Anglicans, the Adventists, the Baptists, the Methodists and the Pentecostalists (Fig. 4).

According to the Pew Forum on Religion and Public Life, there were a total of more than 800 million Protestants in 2010, making up 37% of the global Christian population. They include 300 million in Sub­Saharan Africa, 260 million in the Americas, 140 million in Asia-Pacific region, 100 million in Europe and 2 million in Middle East­ North Africa (Pew Research Center, 2011).

Protestants vary in their beliefs on IVF, and unlike the Catholic Church, there is not one set of ethical guidelines for Protestant couples to follow regarding its use. Those who support IVF, limit its use to married couples. All the embryos must be replaced into the uterus and selective reduction is not allowed.

## Assisted reproduction in the Anglican Church

**Fig. 5:**
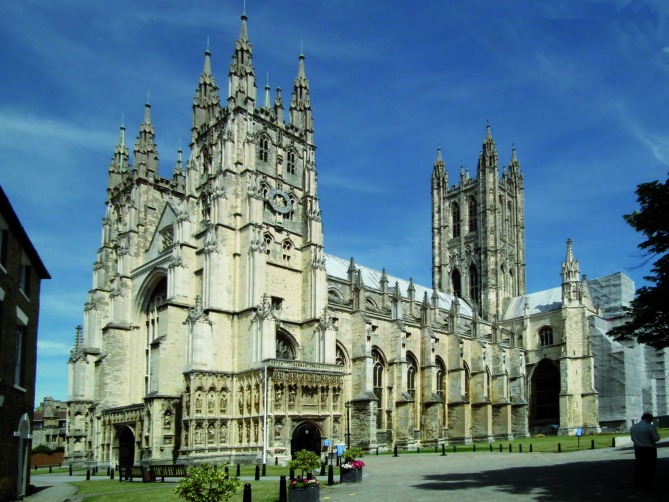
— Canterbury Cathedral in Kent, England, has been the seat of the spiritual head of the Church of England for nearly five centuries. The Church of England has about 85 million followers worldwide.

Following Martin Luther’s reformation, King Henry VIII who already had a personal dispute with the Catholic Church regarding his divorce from Catherine of Aragon, dissolved the Catholic monasteries and abbeys and established the Church of England. The newly­-separated Anglican Church was given some formal structure in 1562 during the reign of Elizabeth I, making the English Monarch the Supreme Governor of the Church of England and the Archbishop of Canterbury the senior bishop and principal leader of the Church and the symbolic head of the worldwide Anglican Communion (Fig. 5). Anglicanism spread as the British colonists settled in North and South America, Africa and Asia. Today, the number of Anglicans in the world is over 85 million. Of those, 36.7 million live in Africa and 26 million in England.

The Anglican Church does not offer a moral status to the embryo and believes that a moral status can only be given to an individual with a well­ established personality (Church of England Board of Social responsibility, 1984; Ron­-El and Rizk, 2012). Consequently, it allows assisted reproductive techniques, IVF and ET and allows the doctors to use sperm obtained after masturbation. The Church has recently accepted gamete donation by third parties, although it stated that individual Anglicans may decide not to use donor gametes.

However, the Church of England expressed concern at offering fertility treatment to single women and gay couples. When the law (Human Fertilization and Embryology HFE act) was discussed in the British House of Lords, Lady Soultan proposed an amendment to the HFE Bill to prevent single women from getting a treatment. Her amendment was defeated by only one vote. The Church of England subsequently stated in September 2012 that “Bringing the care of an adoptive home to a needy child is a wholly different circumstance to deciding in advance to use IVF technology to bring into the world a child who will, ‘by design’, never have a father (or mother, in the case gay men commissioning a child by IVF surrogacy). It sends the signal that everyone has a right to a child and this ‘right’ over­rules consideration of that child’s welfare” (Church of England, 2012).

## Assisted reproduction and the Eastern Orthodox Churches

**Fig. 6:**
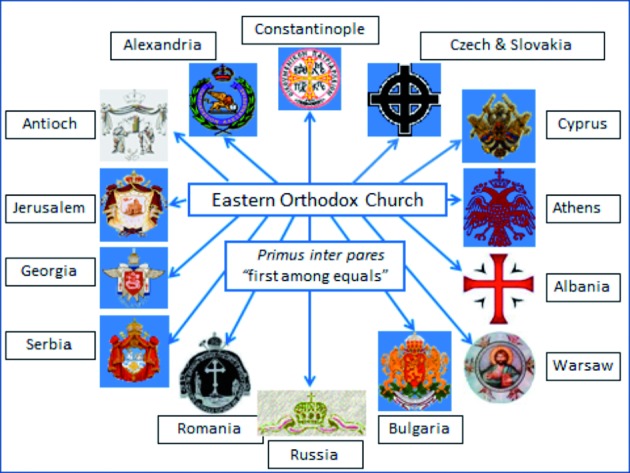
— The 14 major Eastern Orthodox Churches

In 1054, Patriarch Michael Cerularius refused to recognize the Church of Rome’s claim to be the head and mother of all the churches. As Pope Leo IX had died, Cardinal Humbert excommunicated Patriarch Cerularius, while Patriarch Cerularius in return excommunicated Cardinal Humbert. The East–West Schism or The Great Schism resulted, dividing Christianity into the Eastern (Greek) Orthodox Church and the Roman (Latin) Catholic Church. Today there are 14 main Eastern Orthodox Churches, which are considered equal, although the Church of Constantinople has traditionally been considered the mother Church. Each church is headed by a Patriarch who is equal in rank to the other Patriarchs, although the Patriarch of Constantinople is considered *“Primus inter pares”* (first among equals) (Fig. 6).

The Eastern (Greek) Orthodox Church is not as strict as the Roman (Latin) Catholic Church regarding assisted reproduction. It allows the medical and surgical treatment of infertility including IUI using the husband’s sperm but cannot accept IVF and other assisted reproductive techniques, surrogate motherhood, donor insemination and embryo donation. The Church suggests adoption as an alternative to those couples unable to accept their sterility problem. If this is not possible, then the Church could accept fertilization techniques that do not involve surplus embryos, or include any form of donation or embryo destruction. She could also accept assisting the reproduction procedure by using only the parents’ gametes and fertilizing as many embryos as will be implanted (Report of the Bioethics Committee of the Church of Greece, 2005; The Holy Synod of the Church of Greece, 2007).

## Assisted reproduction in the Coptic Church

**Fig. 7:**
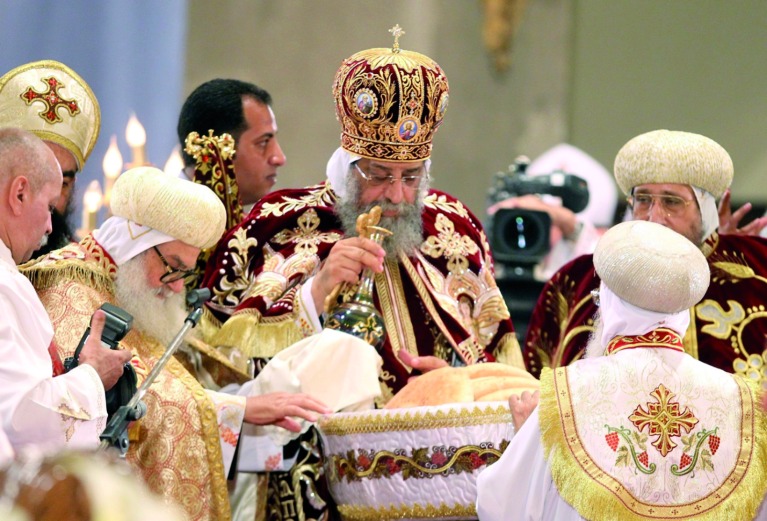
— Pope Tawadros II is 118th Pope of Alexandria and the Patriarch of All Africa on the Holy See of Saint Mark. The Coptic Orthodox Church of Alexandria is the largest Christian Church in Egypt and the largest in the Middle East overall. It has about 15 million followers worldwide.

The Coptic Orthodox Church became a distinct church body since the Council of Chalcedon in AD 451. It traces its roots to St Mark the apostle who preached in Alexandria, Egypt in the early days of Christianity. It has about 15 million followers, who reside mainly in Egypt, although there are many Coptic communities in the USA, Europe and Australia. It is headed by the Pope of Alexandria and is independent from all other Churches (Fig. 7). The Church of Abyssinia (Ethiopia) is also an offshoot of the Coptic Church.

The Coptic Church is more lenient than other Eastern Orthodox Churches in its views on assisted reproduction. According to the late Bishop Gregorios, the Bishop of theological studies, Coptic culture and scientific research, IVF is accepted as long as the oocyte and sperm are taken from the husband and wife, fertilization occurred in vitro with no doubt about gamete mixing. Embryo transfer must be performed to the mother who is the source of the oocytes. Artificial insemination with the husband’s sperm (AIH) is also accepted, but gamete donation is not (Gregorius, 1988; Rizk, 2008).

## Assisted reproduction in other Christian churches

People who belong to other traditions that view themselves as Christians make up about 1% of the global Christian population. With the exception of Christian Scientists, most of these Churches (Baptist, Methodist, Lutheran, Mormon, Presbyterian, Episcopal, United Church of Christ, Jehovah’s Witnesses, Seventh Day Adventists, Mennonites) have more liberal attitudes towards the traditional infertility workup and treatments. All of them accept IVF with spouse’s gametes and no embryo wastage. However, gamete donation is forbidden. On the contrary, the Christian Scientists Church does not condone IVF because of the use of medication and surgical techniques, but leaves the final decision to the individual couples. It also opposes gamete donation and surrogacy but has no objection of artificial insemination using the husband’s sperm (Abbott, 2002; Cohen, 2002).

## Islam and assisted reproduction

**Fig. 8:**
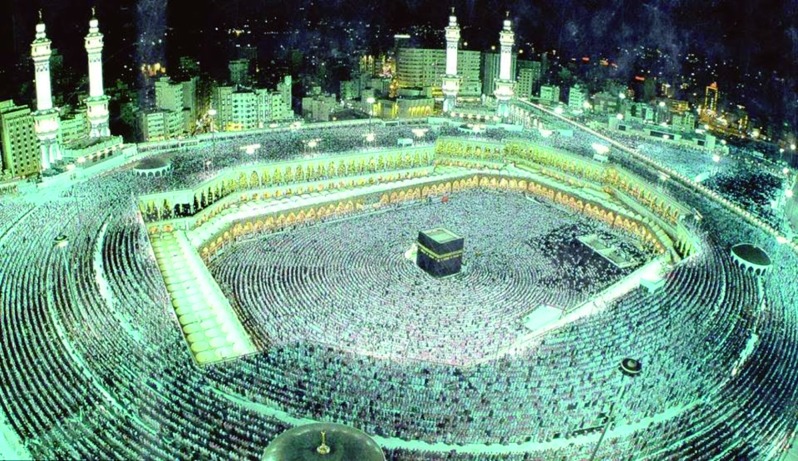
— Each year at the time of the Hajj (Pilgrimage), two million Muslims visit Mecca to perform the ritual, which is one of the five pillars of Islam. Islam is the religion of 1.6 billion people worldwide, 90% of whom follow Sunni Islam.

**Fig. 9:**
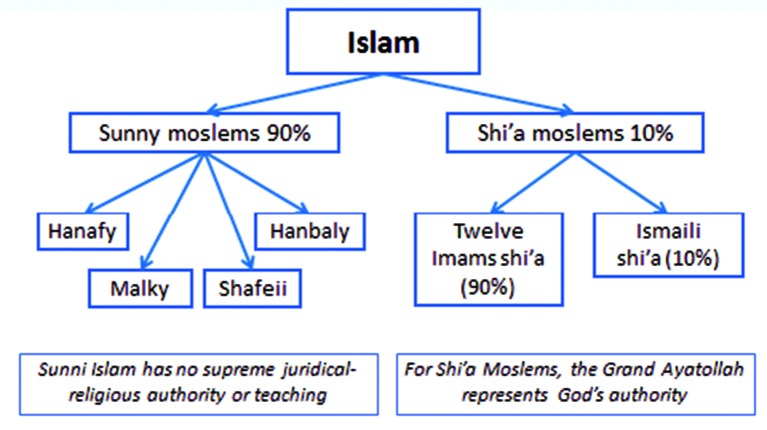
— Main religious sects in Islam.

Muslims (followers of the religion of Islam) form about 22.3% of the world population (1.6 billions). According to the Muslim tradition, Islam is not only a religion, but also a way of life, as the teachings of Islam cover all the fields of human activity: spiritual and material, individual and social, educational and cultural, economic and political, national and international (Serour, 2008) (Fig. 8). Islamic scholars like to highlight the fact that Islam is a religion of ease (Yusr) and not hardship (‘Usr) and that Islamic jurisprudence is based on the following principles: (1) any act is permissible unless prohibited by a text (in the Quran), (2) any act should produce no harm and should not be done under harassment, (3) necessity permits the prohibited, and (4) if two necessary acts are harmful, one should choose the one leading to the lesser harm (Serour, 2008). In addition, Islam affirms the importance of marriage, family formation and procreation (Quran 13:38, 16:27, 42: 49-­50). However, adoption is not permitted but kind upbringing of orphans is encouraged (Quran 32:4­5). Today, Muslims are divided into 2 sects: Sunnis Muslims and Shi’a Muslims (Fig. 9).

## Assisted reproduction in Sunni Islam

Sunni Muslims who form about 90% of Muslims have no single supreme religious leader but have many religious bodies, which issue Fatwas (religious opinions/rulings) on important issues related to everyday activities of their followers. In theory, their opinion is not binding but in practice Sunni Muslims follow the Fatwas issued by those bodies. In addition, the laws of Muslim countries follow these Fatwas in most of instances, particularly in matters of birth, death, marriage and divorce and consequently assisted reproduction. Practitioners in Sunni communities/countries have been following two Fatwas issued on assisted reproduction: (1) a Fatwa from Al­-Azhar Religious Institution based in Cairo, Egypt issued in 1980 and (2) a Fatwa from the Islamic Fikh Council based in Mecca in Saudi Arabia issued in 1984 (Gad­-el-­Hak, 1980; Inhorn, 2006). In addition, two guidelines have been issued on the same subject: (1) Guidelines from the Organization of Islamic Medicine based in Kuwait in 1991 and (2) Guidelines from the Islamic Educational, Scientific and Cultural Organization issued in Rabat, Morocco in 2002 (Serour, 2008).

According to these Fatwas and guidelines of Sunni Islam, all forms of assisted reproduction are allowed as long as the sperm and oocyte are those of the husband and his wife and the embryo is replaced into the wife’s uterus during an existing marriage contract (which ends by death or divorce) i.e. a third party cannot be involved (Serour, 2008). Cryopreservation of spermatozoa and oocytes is also allowed as well as cryopreservation of embryos. These embryos remain the property of the couple and may be transferred to the same wife in a subsequent cycle but only during the validity of the current marriage. Research on embryos younger than 120 days is also allowed as according to the Quran, the “soul” enters the body of the foetus after 120 days. However, during the meeting of Muslim scholars at the ISESCO (Islamic UNESCO) in Rabat in 2002 it has been suggested that research can be done only until the age of 14 days, and that every country should establish a national ethical research committee (Serour, 2008). Foetal reduction is also allowed if the prospect of carrying the pregnancy until viability is small. It is also allowed if the life of the mother is in jeopardy. Sperm and oocyte donation are obviously not allowed and pregnancy after the menopause is not allowed when using donated oocytes. However, pregnancy after the menopause is allowed when using cryopreserved oocytes of the same woman in an existing marriage contract. Therapeutic cloning for regenerative medicine is also allowed, but reproductive cloning is prohibited. Somatic gene therapy is allowed but germ line gene therapy is still debated.

For most Sunni Muslims, surrogacy is not permitted. In reality, the Fatwa from the Fikh Council issued in Mecca in 1984 had allowed surrogacy if the oocyte donor and the surrogate mother are both married to the same man. However, this Fatwa was reversed in 1985 when men started to have second wives just to obtain their oocytes and then divorce them. Recently, a new debate has also opened in favour of permitting surrogacy comparing the surrogate mother to the wet nurse (Serour, 2008). In Sunni Islam, PGD to avoid genetic diseases is allowed and considered better than pre­natal diagnosis and abortion. On the other hand, PGD/sperm selection techniques for sex pre­ selection is not allowed for the first child but a recent Fatwa by Al­-Azhar allowed it for family balancing if the couple already has two children of the same sex (Dar­-Al-­Ifta, 2016).

## Assisted reproduction in Shi’a Islam

**Fig. 10:**
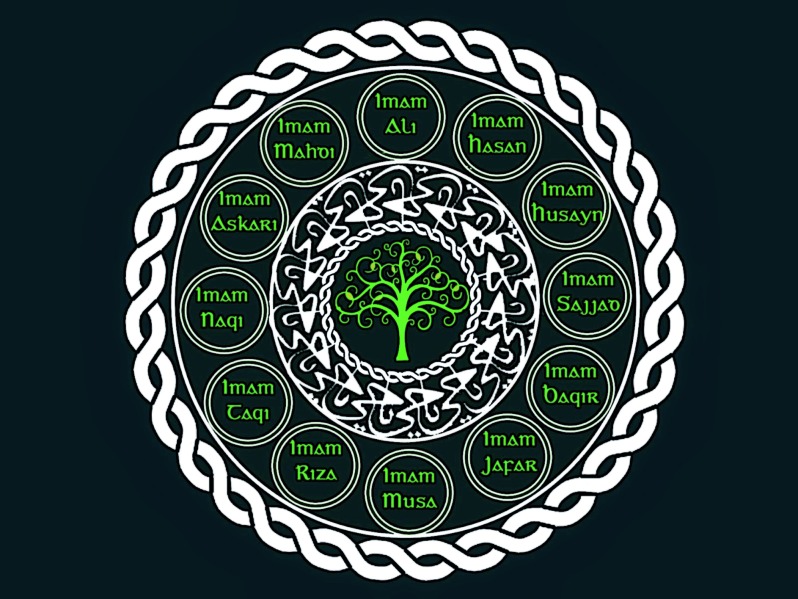
— Ten percent of Muslims worldwide follow Shi’a Islam. Ten percent of Shi’as are “Twelvers” and believe in the 12 Imams who are the bloodline of Prophet Mohamed. They believe that Ali who was Prophet Mohamed’s cousin and son­-in-­law (and the other Imams) should have followed the Prophet as leader of the Muslim community.

Shi’a form about 10% of Muslims. Of these 90% are Twelvers i.e. followers of the 12 divinely chosen, infallible Imams who form the bloodline of the Prophet Mohammed. For them, the last (12th) Imam (Al­-Mahdy) disappeared and will come back at the end of time to spread peace on earth. Until Imam Al­-Mahdy returns, a supreme religious leader is chosen by religious scholars to lead the community of Shi’as and his rulings are sacrosanct (e.g. Ayatollah Khomeini and Ayatollah Khamenei). Twelver Shi’as live mainly in Iran and surrounding countries. The majority of the other 10% of Shi’as are the Ismailis who followed a different bloodline Imam (Ismail) after the 5th Imam. Their current leader is the Agha Khan. They are present mainly in Pakistan and surrounding countries, although many of them emigrated to Europe and the Americas (Fig. 10).

The practice of assisted reproduction in Shi’a Muslim communities follows the Fatwa issued by Ayatollah Ali Hussein Khamenei in the city of Kom in Iran in 1999. The Shi’as principles and practice are similar to the Sunni Fatwas except in one important difference: Shi’as allow gamete donation (Inhorn, 2006). This is because the Sunni Fatwa is based upon the sanctity of the male inheritance line, while the Shi’ite Fatwa is based on the fact that gamete donation does not involve sexual intercourse. Consequently, donor insemination and oocyte donation is allowed by Shi’as with the following consequences: (1) the child of the egg donor has the right to inherit from his (her) biologic mother, (2) the baby born of sperm donation will follow the name of the infertile father rather than the sperm donor, and (3) as with egg donation, the donor child can only inherit from his biological father, the sperm donor, since the infertile father is considered to be like an adoptive father (Inhorn, 2006). Gestational surrogacy is also accepted in Shi’s Islam (Rahmani et al., 2011).

In order to formalize/legalize the practice, Shi’as took advantage of a form of temporary marriage called *mut’aa* (also called *sigheh* in Iran), allowed only in Shi’a Islam. This is a union between an unmarried Muslim woman and a Muslim man, which is contracted for a fixed time period in return for a set amount of money. The couple present before a Shi’a religious court, which determines the necessity of gamete donation. The decision is made in the presence of witnesses, the IVF doctor and with the agreement of both parties (infertile couple and donor). The husband does a *mut’aa* marriage with the donor for the necessary period of time, after which the marriage is dissolved spontaneously (Inhorn, 2006).

However, this arrangement cannot be applied in case of sperm donation as the wife cannot have a second husband. Consequently, second thoughts about sperm donation are now being considered in Shi’a Islam trying to answer the following questions: (1) Should sperm donation be allowed at all in the first place, (2) Should the child follow the name of the infertile father or the sperm donor, (3) Should the child inherit from the infertile father or the sperm donor, (4) Are donor children and their “social” parents related at all, and, if not, can they potentially marry each other, which has implications for proper comportment in domestic life (e.g., bathing, veiling, etc.), (5) Is donation permissible if the donors are anonymous, (6) Should donors be financially rewarded, and (7) Can the wife of an infertile husband temporarily divorce her infertile husband, remarrying him after accepting sperm from a donor? (Inhorn, 2006).

## Assisted reproduction in Hinduism

There are about 1 billion Hindus around the world, representing 15% of the global population. Major traditions within Hinduism include Vaishnavism, which is devoted to worship of the god Vishnu, and Shaivism, organized around worship of the god Shiva. More than 99% of Hindus reside in the Asia­ Pacific region (Pew Research Center, 2012).

**Fig. 11:**
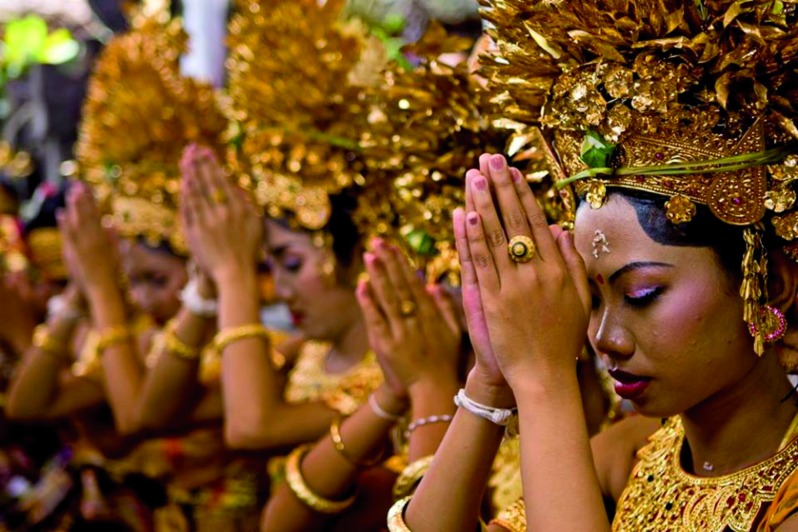
— Hinduism is the religion of the majority of people in India and Nepal. Unlike most other religions, Hinduism has no single founder, no single scripture, and no commonly agreed set of teachings. It has around 1 billion followers around the world.

Hinduism is based in the teachings of the sacred Vedas and Hindu beliefs cover a large range or religious ideologies and philosophic systems starting with polytheism, atheism, pantheism and monotheism. Hindus believe in reincarnation and karma as well as in human’s personal duty called dharma. In the Hindu worldview, the human soul is eternal; it has to live many earthly lives in order to purify itself, to reach perfection and a higher state of existence called “mokasha” (Fig. 11).

Hinduism is a very liberal religion concerning assisted reproduction. In fact the Hindu religion agrees with most of the assisted reproduction techniques, but it demands that the oocyte and the sperm used in the procedure to (better) come from a married couple. However, Hinduism also accepts sperm donation but the donor has to be a close relative of the infertile husband. In addition, abortion is not prohibited and the adoption of a child, which usually comes from a numerous family, is also practiced. This liberal attitude has made India an important destination for reproductive tourism and many couples travel to India for assisted reproduction including members of the LGBT (Lesbians/Gays/Bisexual/Transgender) communities (Storrow, 2006; Sarojini et al., 2011).

## Assisted reproduction in Buddhism

There are about 488 million Buddhists worldwide, representing 7% of the world’s total population. The three major branches of Buddhism in the modern world are Mahayana Buddhism, Theravada Buddhism and Vajrayana (sometimes described as Tibetan) Buddhism. Mahayana Buddhism is widely believed to be the largest and is prevalent in China, Japan, South Korea and Vietnam. Theravada Buddhism, the second­-largest branch, is concentrated in Thailand, Burma (Myanmar), Sri Lanka, Laos and Cambodia. Vajrayana Buddhism, the smallest of the three major branches, is concentrated in Tibet, Nepal, Bhutan and Mongolia. Buddhism began in Asia, and the vast majority of all Buddhists (nearly 99%) still live in the Asia­ Pacific region. Only two other regions – North America (3.9 million) and Europe (1.3 million) – have more than 1 million Buddhists.

**Fig. 12:**
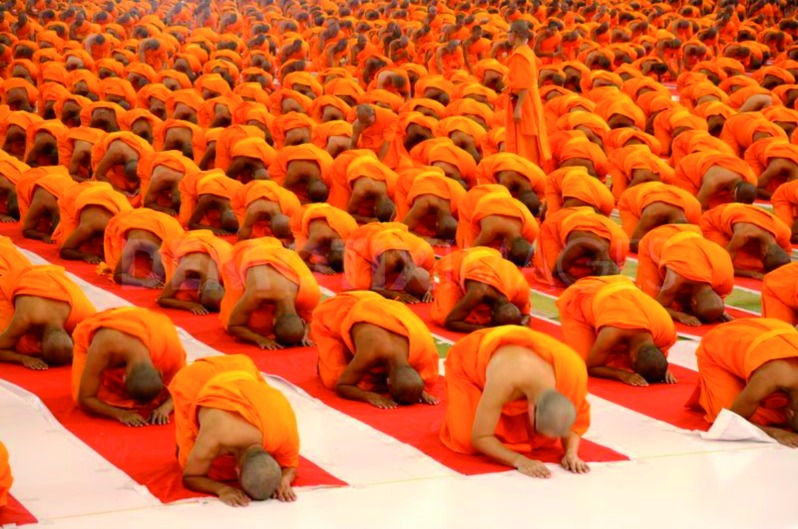
— Buddhism is a non-theistic religion or philosophy (in Sanskrit = dharma) that encompasses a variety of traditions, beliefs and spiritual practices largely based on teachings attributed to Gautama Sidharta, commonly known as the Buddha (the enlightened one).

Buddhism emerged in India around 500 BC and it is based on the teachings of Siddhartha Gautama also known as The Buddha, the enlightened one. Buddhist philosophic system involves a long string of reincarnations and the final purification of the soul and its elevation and entrance in a superior state of existence called “Nirvana” (Fig. 12).

Buddhism is also a very liberal religion regarding assisted reproduction. It allows the use of IVF without restricting the access to this medical procedure to the married couples and sperm donation is also permitted. In the Buddhist tradition, a child conceived from donated genetic material has the right to meet his genetic parents as he reaches maturity (Ying et al., 2015).

## Assisted reproduction in Japanese culture

**Fig. 13:**
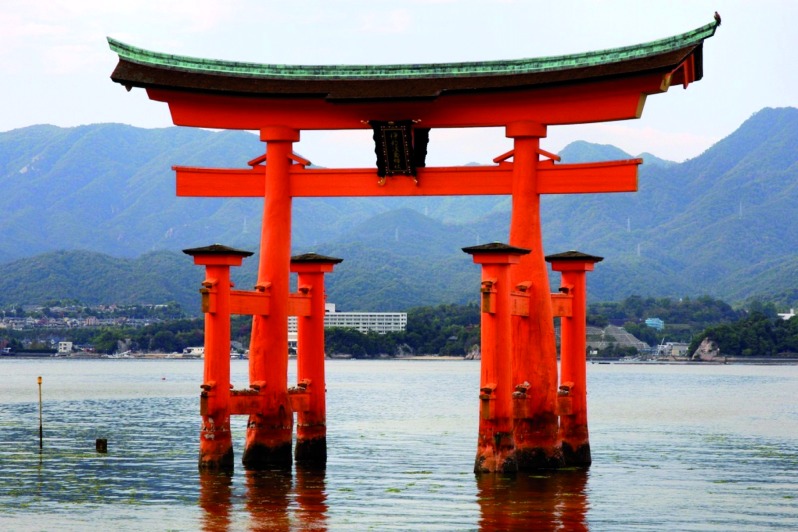
— A Shinto shrine. The Shinto religion also called kami­no­michi, is the ethnic religion of the people of Japan. It focuses on ritual practices to be carried out diligently, to establish a connection between present­-day Japan and its ancient past.

Japanese people can be adherents of Buddhism, Shintoism or both religions. Shintoism is a Japanese faith that has been part of religious life in Japan for many centuries. Although Shinto rituals are widely practiced in Japan, only a minority of the Japanese population identifies with Shintoism in surveys (Fig. 13). The World Religion Database estimates there are almost 3 million Shintoists worldwide, with the vast majority concentrated in Japan. Other new religions practiced in Japan include the Tenrikyo, the Shinreikyo, the Mahakari, the Omoto and PL Kyodan (Pew Research Center, 2015).

Taking all these religions in consideration, the law in Japan allows IUI, IVF and ICSI. Donor insemination is also permitted and the first sperm bank in Japan was established in Tokyo in 1965. However, oocyte donation and surrogacy are forbidden by law. IVF is also prohibited if both partners are infected with HIV (Takahashi et al., 2012).

## Assisted reproduction in Chinese culture

**Fig. 14:**
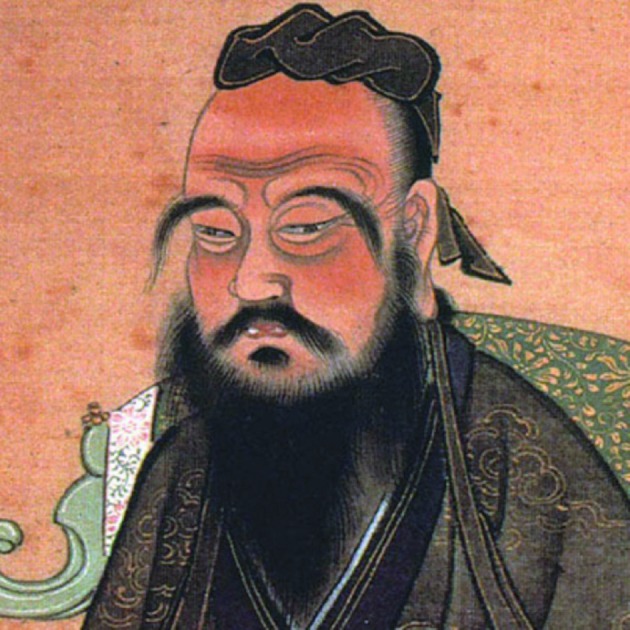
— Confucius (551 ­- 479 B.C.E.), the Chinese thinker and social philosopher, whose teachings and philosophy have deeply influenced people in China, Korea, Japan and Vietnam.

Confucianism, Taoism and Buddhism, constitute the three schools of thought, which have historically shaped the Chinese culture (Fig. 14). Elements of these three belief systems are also incorporated into folk or popular religions. As in Japanese religions, Chinese religions are family­-oriented and do not demand exclusive adherence, allowing the practice or belief of several at the same time. Some scholars prefer not to use the term “religion” in reference to belief systems in China, and suggest “cultural practices”, “thought systems” or “philosophies” as more appropriate terms (Taylor, 1982).

Confucius (551 ­- 479 B.C.E.) was a Chinese thinker and social philosopher, whose teachings and philosophy have deeply influenced Chinese, Korean, Japanese and Vietnamese thought and life. His philosophy emphasized personal and governmental morality, correctness of social relationships, justice and sincerity. Taoism is said to have been founded in the 6th century B.C.E. by Chinese philosopher Lao Tzu and its adherents live predominantly in China and Taiwan. The World Religion Database estimates there are about 8.7 million Taoists.

The practice of IUI, IVF, ICSI, cryopreservation and PGD are allowed in China, but the following procedures are prohibited (1) sex selection without medical indication, (2) surrogate motherhood, (3) embryo donation, (4) gamete donation and (5) human reproductive cloning (Qiao and Feng, 2014).

## Assisted reproduction and adherents of folk religions

**Fig. 15:**
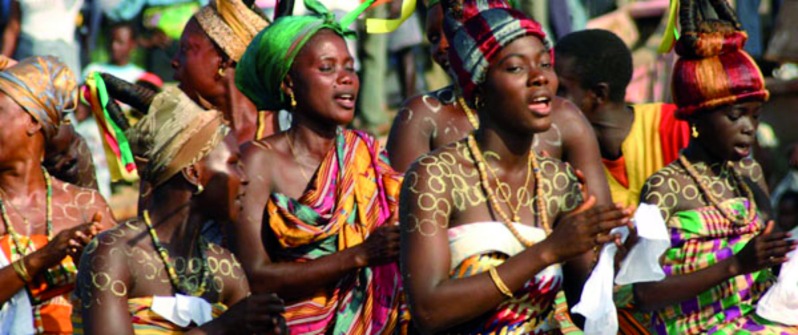
— Practitioners of traditional religions in Sub­-Saharan Africa are distributed among 43 countries, and are estimated to number over 100 million. Traditional African religions are also practiced in the diaspora in the Americas and include Candomble, Santeria, and Vodou.

An estimated 405 million people – or about 6% of the world’s population – are adherents of folk or traditional religions (Fig. 15). Folk or traditional religions are faiths closely associated with a particular group of people, ethnicity or tribe. They often have no formal creeds or sacred texts. Examples of folk religions include African traditional religions, Chinese folk religions, Native American religions and Australian aboriginal religions. Despite the fact that they make the fifth largest religious group in the world, adherents of folk religion are poorly organized and are forced to obey the law of the land whether they live in Africa, China, North and South America or Australia.

## Conclusion

Religion plays a major role in people’s attitudes towards assisted reproduction and various religions have reacted to this treatment in different ways. These range from total acceptance to total rejection of all techniques of assisted reproduction, with many shades in between. Table III summarizes the various assisted reproduction techniques allowed by various religions. People, communities and countries will continue debating the issue as long as more advances are made in assisted reproduction (Halvaei et al., 2014).

**Table III:** — Summary of assisted reproduction technique allowed by various religions/cultures.

	IUI	IVF/ICSI	PGD	Surrogacy	Gamete donation	Foetal reduction
Catholic	No	No	No	No	No	No
Orthodox	Yes	No	No	No	No	No
Protestants	Yes	Yes	No	No	No	No
Anglicans	Yes	Yes	No	No	No	No
Coptic	Yes	Yes	Yes	No	No	No
Judaism	Yes	Yes	Yes	Yes	Yes	Yes
Sunni Islam	Yes	Yes	Yes	Debating	No	Yes
Shi’a Islam	Yes	Yes	Yes	Yes	Yes	Yes
Hinduism	Yes	Yes	Yes	Yes	Yes	Yes
Buddhism	Yes	Yes	Yes	Yes	Yes	Yes
Japan	Yes	Yes	Yes	No	Sperm only	Yes
China	Yes	Yes	Yes	No	No	Yes

## References

[1] Abbot D (2002). The Christian Science Tradition – religious beliefs and healthcare decisions. The Park Ridge Center for the Study of Health, Faith and Ethics.

[2] Abou­-Abdallah M, Rizk Botros, Garcia-Velasco Juan, Sallam Hassan, Makrigiannakis Antonis (2008). The Vatican view of human procreation. Infertility and Assisted Reproduction.

[3] Bishop Gregorios HG (1988). The Christian Opinion. In vitro Fertilization and Embryo Transfer.

[4] (1984). Evidence to the DHSS Inquiry into Human Fertilization and Embryology, 24 February 1984. https://www.churchofengland.org/media/45709/gamete.pdf.

[5] (2012). Response from the Church of England Mission and Public Affairs Council to the Call for Evidence from the Joint Committee on the Draft Human Tissue and Embryos Bill. https://www.churchofengland.org/media/45705/humantissue.pdf.

[6] Cohen S (2002). Protestant Perspectives on the Uses of the New Reproductive Technologies.. Fordham Urban Law Journal.

[7] Gad El­-Hak AGH (1980). Fatwa regarding in vitro fertilization and test tube babies.. Dar El Iftaa Cairo Egypt.

[8] Hackett C, Stonawski M, Potancokova M (2015). The future size of religiously affiliated and unaffiliated populations.. Demographic Research.

[9] Halvaei I, Khalili MA, Ghasemi­-Esmailabad S (2014). Zoroastrians support oocyte and embryo donation program for infertile couples.. J Reprod Infertil.

[10] Holy Bible Genesis 1: 28.

[11] Holy Bible Genesis 30: 14.

[12] Holy Bible Genesis 30: 23.

[13] Holy Quran 13:38.

[14] Holy Quran 16:27.

[15] Holy Quran 42:49-50.

[16] Holy Quran 32:45.

[17] Inhorn M (2006). Making Muslim babies: IVF and gamete donation in Sunni versus Shi’a Islam.. Cult Med Psychiatry.

[18] Kahn SM (2000). Reproducing Jews: A Cultural Account of Assisted Conception in Israel.

[19] (2011). Global Christianity – A Report on the Size and Distribution of the World’s Christian Population.

[20] (2012). “The Global Religious Landscape”. http://www.pewforum.org/2012/12/18/global-religious-landscape-exec/.

[21] (2015). “The Future of World Religions: Population Growth Projections, 2010­-2050”. http://www.pewforum.org/2015/04/02/religious-projections-2010-2050.

[22] Pope Pius XII (1956). Disclosure to those taking part in the Second Naples World Congress on Fertility and Human Sterility. AAS.

[23] Paul (1968). “Humanae Vitae: Encyclical of Pope Paul VI on the Regulation of Birth, sec 12”.

[24] Qiao J, Feng HL (2014). Assisted reproductive technology in China: compliance and non-­compliance. Transl Pediat.

[25] Rahmani A, Sattarzadeh N, Gholizadeh L (2011). Gestational surrogacy: Viewpoint of Iranian infertile women.. J Hum Reprod Sci.

[26] Report of the Bioethics Committee of the Church of Greece.

[27] Rizk Botros, Garcia-Velasco Juan, Sallam Hassan, Antonis Makrigiannakis (2008). The views of the Coptic Orthodox Church on the treatment of infertility, assisted reproduction and cloning. Infertility and Assisted Reproduction.

[28] (1987). Report entitled “Respect for Human Life and the Dignity of Procreation”.

[29] Ron­-El R, Rizk B, Gardner DK, Weissman A, Howles CM, Shoham Z (2012). Religious perspectives in human reproduc­tion. In ’Textbook of Assisted Reproductive Techniques’, Fourth Edition.

[30] Sarojini N, Marwah V, Shenoi A (2011). Globalisation of birth markets: a case study of assisted reproductive technologies in India.. Global Health.

[31] Schenker JG (2005). Assisted reproductive practice: religious perspectives.. RBM Online.

[32] Serour G, Rizk B, Garcia-Velasco J, Sallam H, Makrigiannakis A (2008). Islamic perspectives of ethical issues in ART.. Infertility and Assisted Reproduction.

[33] Silber S, Rizk B, Garcia-Velasco J, Sallam H, Makrigiannakis A (Cambridge University Press). Infertility, IVF and Judaism.. Infertility and Assisted Reproduction.

[34] Stonawski M, Skirbekk V, Hackett C (2015). Global population projections by religion: 2010­-2050. Yearbook of International Religious Demography.

[35] Storrow RF (2006). Quests for Conception: Fertility Tourists, Globalization and Feminist Legal Theory.. Hastings Law Journal.

[36] Takahashi S, Fujita M, Fujimoto A (2012). The decision­making process for the fate of frozen embryos by Japanese infertile women: a qualitative study.. BMC Med Ethics.

[37] Taylor RL (1982). Proposition and Praxis: The Dilemma of Neo­ Confucian Syncretism. Philosophy East and West.

[38] (2007). Bioethics Committee, Basic positions on the ethics of Assisted Reproduction, Athens, p.

[39] Wahrzman M Jewish Virtual Library.

[40] (2016). Wikipedia.

[41] Ying L­Y, Har L, Loke A (2015). The experience of Chinese couples undergoing in vitro fertilization treatment: perception of the treatment process and partner support.. PLoS One.

